# No evidence of the effect of cognitive load on self-paced cycling performance

**DOI:** 10.1371/journal.pone.0217825

**Published:** 2019-05-31

**Authors:** Darías Holgado, Mikel Zabala, Daniel Sanabria

**Affiliations:** 1 Department of Physical Education and Sport, Faculty of Sport Sciences, University of Granada, Granada, Spain; 2 Mind, Brain and Behaviour Research Centre, Department of Experimental Psychology, University of Granada, Granada, Spain; University of Essex, UNITED KINGDOM

## Abstract

**Objectives:**

To test the hypothesis that cognitive load (low vs. high load) during a 20 min self-paced cycling exercise affects physical performance.

**Design:**

A pre-registered (https://osf.io/qept5/), randomized, within-subject design experiment.

**Methods:**

28 trained and experienced male cyclists completed a 20 min self-paced cycling time-trial exercise in two separate sessions, corresponding to two working memory load conditions: 1-back or 2-back. We measured power output, heart rate, RPE and mental fatigue.

**Results:**

Bayes analyses revealed extreme evidence for the 2-back task being more demanding than the 1-back task, both in terms of accuracy (BF_10_ = 4490) and reaction time (BF = 1316). The data only showed anecdotal evidence for the alternative hypothesis for the power output (BF_10_ = 1.52), moderate evidence for the null hypothesis for the heart rate (BF_10_ = 0.172), anecdotal evidence for RPE (BF_10_ = 0.72) and anecdotal evidence for mental fatigue (BF_10_ = 0.588).

**Conclusions:**

Our data **seem to** challenge the idea that self-paced exercise is regulated by top-down processing, given that we did not show clear evidence of exercise impairment (at the physical, physiological and subjective levels) in the high cognitive load condition task with respect to the low working memory load condition. The involvement of top-down processing in self-pacing the physical effort, however, cannot be totally discarded. Factors like the duration of the physical and cognitive tasks, the potential influence of dual-tasking, and the participants’ level of expertise, should be taken into account in future attempts to investigate the role of top-down processing in self-paced exercise.

## Introduction

Self-paced exercise is a goal-directed behavior that has been related to both bottom-up [1,2] and top-down processing [3]. Self-paced exercise or pacing refers to a physical activity (e.g., a cycling time trial or a marathon), in which the effort has to be evenly distributed in order to achieve the objective of the exercise (to complete a given distance as fast as possible, or to complete the maximum possible distance in a given time), but without reaching premature exhaustion, based on previous experiences and the perceived duration/distance to cover [4]. Thus, self-paced exercise might be seen as an effortful cognitive task involving body motion that places high demands on the brain and request top-down processing [5], for it involves goal monitoring, cognitive control etc. For example, during a cycling time-trial, cyclists have to make continuous decisions to adapt the pace according to the demands of the event [6], to focus their attention toward relevant stimuli [7] and to inhibit the urge of slowing down [8]. Indeed, Sport Scientists are beginning to recognize the brain and cognitive functioning (e.g., inhibitory control, working memory) as decisive in the control of exercise, in particular self-paced aerobic exercise [4]. Interestingly, the few neuroimaging studies testing participants while exercising have shown activation of the prefrontal cortex, together with the expected sensorio-motor recruitment [9,10], which reinforces the hypothesis of the crucial role of top-down processing on self-paced exercise.

The above-mentioned research is in line with the cognitive resource theory or Reticular-Activating Hypofrontality [11]. This theory proposes that humans have a limited set of metabolic resources and when these resources are shared amongst several tasks, interference occurs between each other [11]. However, other authors have questioned that metabolic resources are a limiting factor and they indicate that the impairment might be rather due to the attentional limitations when perform both tasks [12,13]. Nonetheless, there is evidence showing that concurrent exercise (which might tap the same underlying cognitive processes) can impair cognitive performance compared to when the cognitive a task is performed alone [14–16]. For instance, Epling et al. [17] found a decrement in the number of words recalled when participants completed a 5 min self-paced (outdoor) running exercise.

To date, in the majority of studies interested on the interplay between top-down cognitive processing (cognitive load) and exercise performance, participants performed the cognitive task prior to the exercise session (c.f. Van Cutsem et al. [18]; Pageaux & Lepers [19]). Little is known regarding the impact of top-down processing (cognitive load) during self-paced exercise. For example, Blakely et al., [20] examined the impact of cognitive load (tone counting with two levels of difficulty) on a group running on an even surface and in a group running on an uneven (trail) surface. They found a linear trend in both groups for a worsening performance with increasing of difficulty of the cognitive task and also higher reports of workload, task-focus and feelings of being mentally exhausted with the increased cognitive load. Similarly, Malcolm et al. [21] found that the increase in cognitive load while participants walked in a treadmill, modified the gait pattern during the more challenging task compared to the control condition. Likewise, Daniel and Newell [22] evaluated the influence of solving a mental arithmetic task with two levels of difficulty (easy and hard, and with respect to a control condition without mental task) on the walk-run transition speed. Authors hypothesized that the walk-run transition would occur later while participants solved the mental arithmetic because the distraction created by focusing on the math task would mitigate perception of effort that contribute to triggering the switch to running. Despite the run-walk transition speed occurred later compared to the control condition (i.e., participants started to run later, although walking at higher speed would require higher capacity of attention), they did not find difference between the easy and the hard (cognitive) condition.

Here, we provide novel evidence on self-pacing during (cycling) exercise under two different conditions of cognitive load. We chose a n-back task with two levels of cognitive demands (low 1-back and high 2-back) during cycling self-paced exercise. The n-back task has been shown to involve both inhibitory control and working memory [23,24]. Therefore, performing an executive task with low or high demands would affect self-paced exercise performance, if self-paced exercise depends on top-down processing. In particular, we expected lower performance and increased perceived effort during the high-load condition than during the low-load condition. By including two conditions with different levels of cognitive (top-down) load, we tried to ensure that any effect on physical performance would be a consequence of the cognitive load and not the effect of performing a single task compared to dual-tasking. In others words, finding impaired exercise performance in a dual-task (cognitive + physical) compared to a single (physical) task could point to both an effect of cognitive load or to the mere effect of dual-tasking (irrespective of the level of cognitive load). Note that this is common practice in the literature that investigates the effects of physical load on cognitive performance (i.e., physical demands are manipulated while the cognitive task remains the same [25–27]). And, more importantly, in the previous studies investigating the impact of mental fatigue on subsequent physical performance [28]. In sum, our study tests the hypothesis that self-pacing effort during exercise rely on top-down processing.

## Material and methods

### Design

A randomized, counterbalanced, within-participant procedure was carried out. This study was approved by the University of Granada Ethics Committee (287/CEIH/2017). All experimental procedures have been designed to comply with the Declaration of Helsinki. Before being recruited to the study, participants read and signed the informed consent. All data was entered in a case report form and subsequently in a computerized database. Participants were naïve to the aim of the study in order to avoid an expectation effect. Once they completed their participation, they were debriefed with the purpose of the study. We pre-registered the methods and planned analyses of this study on the Open Science Framework. This was done on April 4, 2018, and can be found at https://osf.io/qept5/registrations/ together with the raw data files.

### Participants

Participants were recruited by local advertisements in the Granada area, in Spain. Experimental sessions took place in the Faculty of Sport Sciences, in the University of Granada, Spain. Only male trained cyclists with a reported weekly training of more than 6h/week and between 18 to 40 years were included in the study. We decided to include only trained (although not elite) cyclists because they are used to performing bouts of self-paced exercise at the highest possible intensity. Untrained individuals might stop exercising because of the inability to sustained the effort, discomfort due to the body posture on the ergometer or any related factors. Exclusion criteria were the presence of symptomatic cardiomyopathy, metabolic disorders such as obesity (BMI >30) or diabetes, chronic obstructive pulmonary disease, epilepsy, therapy with b-blockers and medications that would alter cardiovascular function, hormonal therapy, smoking, and neurological disorders.

The sample size was determined following a Bayesian approach. We planned to collect data from a minimum of 20 participants. Then, we monitored the Bayes factor (for the average power output) and stopped the experiment whenever the Bayes factor reached a moderate evidence to either support (BF > 6) or to reject the null hypothesis (BF < 1/6). We also planned to stop the experiment when we reached the maximum number of participants (40 participants) which we expected to be able to recruit. In any case, even if we were not able to reach the 40 participants sample, we would stop the experiment on June 30th, 2018 when the academic year ends. A final sample of 28 male trained cyclists’ participants completed the study (27.03 ± 7.41 years).

### Apparatus and materials

We used an indoor cycling trainer Phantom 5 ergometer (CyleOps, Madison, USA) to conduct the experimental self-paced exercise. The Phantom 5 measures the power output using an onboard power meter PowerTap (PowerTap, Madison, USA) with power accuracy of +/- 1.5%. We used the Rouvy app to monitor and record power output and heart rate (HR) through a sensor band attached to the participant’s chest (SmartLab, Dossenheim, Germany) during the experiments. We used a PC and the E-Prime software (Psychology Software Tools, Pittsburgh, PA, USA) to control the stimulus presentation and response collection for the n-back task. The center of the PC screen was situated at 100 cm (approx.) from the participants’ head and at his eye level.

### Procedure

#### Screening visit

The first day of the study, participants attended to the Faculty of Sport Sciences for a familiarization visit. After verifying that the participants met the inclusion criteria, participants performed the two levels of the n-back task (1-back and 2-back) (counterbalanced across participants) and a 10-minutes self-paced exercise. The aim of this session was that of familiarize the participants with the lab and cycle ergometer. Participants were experienced cyclists and used to performing self-paced effort such as the 20 min self-paced cycling test.

#### Experimental sessions

After the familiarization visit, participants attended to the lab in two separate visits to perform the 20 min self-paced cycling exercise and the n-back task during exercise. Participants were asked to refrain from drinking alcohol (48 h abstinence) and instructed not to perform any exhaustive exercise in the 48 h before the experimental session. Upon the arrival at the laboratory, participants performed the 20 min self-paced exercise preceded by 10 min warm-up in the ergometer. Participants were instructed to achieve the highest average power output (watts) during the self-paced exercise and perform the n-back task as accurately as possible (see below n-back task). They were allowed to modify the power load during the exercise by pressing two buttons attached to the handlebar (± 10 watts) on the side of their non-dominant hand. Participants were aware of the elapsed time (helping them to self-regulated the effort), but they were blinded to performance variables (watts and heart rate) during the self-paced exercise. The experimenter did not intervene (e.g., encouraging participants) during the test. Participants were instructed to do their best in both tasks, the self-paced exercise and the cognitive task.

#### N-back task

Participants completed a 1-back and a 2-back task (counterbalanced across participants). One of four digits (1, 2, 3 or 4; 2.67° x 1.53°; 2.67° x 1.62°; 2.67°x 1.62° and 2.67°x 1.81°, respectively) was presented for 500 ms, followed by a fixed delay of 2500 ms (see Luque-Casado, et al., 2015 [29] for a similar procedure). In the 2-back condition, participants had to respond, at any time during the presentation of the stimulus or the delay period, only when the current stimulus was the same as the stimulus presented two trials before. If the stimulus on the screen matched the stimulus presented two trials before, the participant had to press a USB button attached to the handlebar of the ergometer on the side of their dominant hand. Otherwise, the participants had to withhold the response. A new stimulus was presented every 3000 ms (i.e., 500 ms of stimulus presentation and 2500 ms of fixed delay). For the 1-back condition the procedure was similar of that of the 2-back, but they had to respond only when the current stimulus was the same as the previous stimulus. The digit appearing on each trial was randomly selected, which means that, on average, the current digit was the same as the one presented one or two trials earlier in 25% of the trials. There was not feedback after each trial. The overall duration of the task was 20 min, divided in 4 blocks. At the end of each block participants rated their perceived exertion. The N-back task was reset after each block. Participants completed the first minute of the self-paced exercise without the task so that they could increase the power output load. For each stimulus, the response accuracy (percentage of correct responses) and reaction time were recorded. Accuracy was stressed over response speed.

#### Subjective scales

We assessed the subjective mental workload of both the 1-back and 2-back sessions with a visual analog scale (VAS). We used a VAS ranging from 0 (low) to 100 (high) in response to the following question before and after each experimental session: “What is your mental fatigue level now?” [30].

Rate of perceived exertion (RPE): we asked the participant to rate their perceived effort in the 6–20 RPE [31] scale after each block of the n-back during the test (i.e., 4 time points). The scale appeared in the screen for 10 seconds and they rated the perceived exertion. All participants were familiarized with the scale, since they had already participated in previous (similar) studies. Nonetheless, we stressed that they should only rate the feeling of effort experienced during the physical exercise, not during the cognitive task.

### Statistical analysis

#### Confirmatory analysis

We calculated the default Bayes factor for a paired, one-sided t-test (2-back vs 1-back) using the open-source JASP statistical package. We used a one-sided Bayesian hypothesis test with a prior of d = 0.4 for (small-medium) effect size on the alternative hypothesis to quantify the evidence for the hypothesis that the high cognitive load induced by the 2-back task would impair the performance in the self-paced exercise with respect to the 1-back condition. Note that in the pre-registration form, we incorrectly stated the we would use a Cauchy prior r = 1 as effect size.

The dependent variables for the self-paced exercise were the average power output, the average heart rate and RPE. The dependent variable for the n-back task was the global accuracy. We used the VAS to check the task demands of n-back after each experimental session. It was analyzed by (normalized) rating change: post-test rating minus pre-test rating divided by post-test ratting plus pre-test rating.

#### Exploratory analysis

We tested if self-paced exercise performance and RPE during the cognitive task varied as a function of time by including the variable block in the analysis (i.e., four blocks of 5 min).

Furthermore, we correlated the effect of the intervention (difference of power output between the low and high cognitive load) with the effect of the cognitive load (difference of accuracy in the n-back task between low and high load condition).

Even if we did not mention it in the pre-registration form, we also analyzed the reaction time from the n-back task following the recommendation of the Reviewers of a previous version of this article. Note, though, that we considered the accuracy in the n-back task as the main manipulation check.

## Results

### Confirmatory analysis

#### N-back task

The Bayes factor for the accuracy in the n-back task during the self-paced exercise was BF_10_ = 4490, indicating that the observed data are more likely under the alternative hypothesis that indicate the presence of effect. According to the classification system proposed by Jeffrey [32], this represents an extreme evidence for the alternative hypothesis that there is a difference on accuracy in the low and in the high cognitive load. The accuracy (percentage of correct responses) for both conditions were: 0.96 (95% Credible Interval (CI) 0.92–0.98) and 0.88 (95% CI 0.85–0.91) for the low and the high cognitive load conditions, respectively.

#### Performance

**Fig 1:**
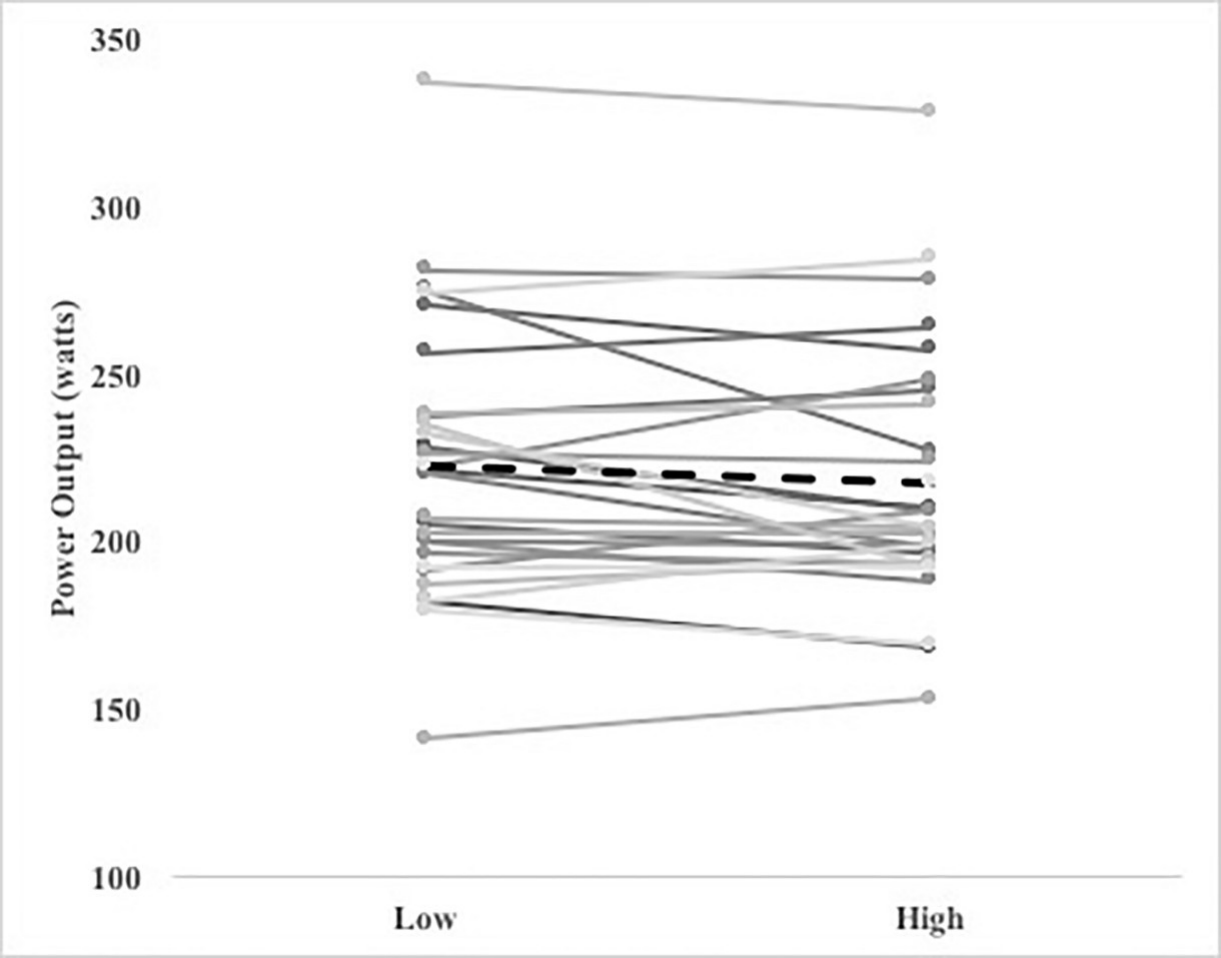
Power output (watts) profile. Power output for each participant and condition during the 20’ self-paced exercise. Dashed line represents the overall mean.

The Bayes factor for the average power output of self-paced exercise was BF_10_ = 1.524, indicating that the observed data are more likely under the alternative hypothesis. However, this represents an anecdotal evidence for the alternative hypothesis that there is a difference on physical performance between the low and the high cognitive load. The average power output for both conditions were: 222 (95%CI 206.4–237.6) watts, and 217 (95%CI 201.9–232.1) watts for the low and the high cognitive load conditions, respectively (Fig 1).

The Bayes factor for the average heart rate during the self-paced exercise was BF_10_ = 0.172, indicating that observed data are more likely under the null hypothesis. The BF indicated a strong evidence in favor of the null hypothesis that the average heart rate during the exercise was not different between both conditions. The heart rate for both conditions were: 159 (95%CI 154–163) bpm and 160 (95%CI 156–165) bpm for the low and the high cognitive load conditions, respectively.

The Bayes factor for the average RPE in the self-paced exercise was BF_10_ = 0.720, which indicate an anecdotal evidence in favor of the null hypothesis indicating the absence of effect between low and high load conditions. The average RPE for both conditions were: 14.88 (95%CI 14.2–15.5) and 15 (95%CI 14.45–15.65) for the low and the high cognitive load conditions, respectively.

The Bayes factor for the normalized VAS score after the self-paced exercise was BF_10_ = 0.588. The Bayes factor supports the null hypothesis and yield anecdotal evidence in favor of the null hypothesis. The normalized VAS score for both conditions were: 0.31 (95%CI 0.15–0.47) IU and 0.37 (95%CI 0.21–0.54) IU for the low and the high cognitive load conditions, respectively.

#### Exploratory analysis

To explore our data further in order to find an alternative explanation (to that of the absence of a true effect of cognitive load on physical performance) to our null finding, we performed additional exploratory analyses.

The results showed that there was strong evidence against the interaction Cognitive load x Block (i.e., stronger evidence for the model without interaction) for the power output in the self-paced exercise, BF_10_ = 0.085. Similarly, the results yielded that there was a strong evidence against the interaction for the RPE in the self-paced exercise BF_10_ = 0.093.

The results showed that there was not a correlation between difference in the power output and the difference in accuracy in the n-back task, r = 0.022, BF_10_ = 0.381.

The Bayes factor for the reaction time in the n-back task was BF_10_ = 1316 and also represented extreme evidence for the alternative hypothesis that indicate the presence of an effect. The reaction times for both conditions were 614.8 (95%CI 549.1–680.4) ms and 801.1 (95%CI 709.9–892.3) ms for the low and the high cognitive load conditions, respectively.

## Discussion

The purpose of this study was to test the hypothesis that executive (cognitive) load would interfere with exercise performance in a 20 min cycling self-paced exercise. The results of the accuracy (and reaction time) in the cognitive task suggest that the high cognitive load condition was more demanding than the low cognitive load. However, despite of the increased executive (cognitive) demands, participants did not seem to impair their physical performance or changed the perceived exertion. In turn, the difference in cognitive performance between the high and low cognitive load conditions cannot be explained by difference in physical performance (at the objective and subjective levels). The results only provided anecdotal evidence for the alternative hypothesis that high cognitive load during exercise might affect physical performance. However, heart rate and RPE data showed moderate to anecdotal evidence in support of the null hypothesis. Moreover, the exploratory analysis also indicated that the effect did not vary as a function of time, i.e., the power output, heart rate and RPE were similar across conditions and time. Finally, the VAS question: What is your mental fatigue level now?” only showed anecdotal evidence for the null hypothesis. During the process of reviewing this manuscript, Reviewers noted that the question we asked to participants could not address correctly the cognitive load of the task. Even if this could be interpreted as a limitation for this study, the purpose was only to obtain an additional measure of cognitive load during the n-back task. In any case, as we have previously mentioned, the results of the accuracy and reaction time data from the n-back task clearly showed a higher cognitive load for the 2-back compared to the 1-back task.

Self-paced exercise might be considered an effortful cognitive task which require the activation of brain areas related to goal monitoring, cognitive control, etc. It has been proposed that self-paced exercise is continuously monitored by feedforward and feedback between peripheral systems and the brain [33]. However, in most of the related studies the athletes have performed the cognitive task prior to exercise [34] and little is known about the effect of performing a challenging cognitive task during a cycling self-paced exercise. To date, we have only found similarities to cycling exercise in studies investigating gait/running pattern. Indeed, gait is also an attention-demanding task that, according to some authors [35], demands high levels of executive processing and memory. When participants perform a cognitive task during the gait, they seem to alter the normal walk pattern to counter the demands of the cognitive task [21,22]. Although gait and cycling might be considered similar tasks (i.e., cyclical and continuous activities), we cannot extrapolate these results to our experiment.

The logic of our study was straightforward: if self-paced exercise is regulated by top-down processing and the n-back also requires top-down processing [24], it was plausible to expect an impairment in physical performance due to the inability to self-pace efficiently. Moreover, we expected that participants would perceive the physical effort harder in the high cognitive load condition than in the low cognitive load condition. The analysis showed anecdotal evidence for the null hypothesis i.e., no effect of cognitive load at subjective levels of physical performance, than for the alternative hypothesis and it similar to previous studies in which the cognitive load did not affect RPE during exercise [36]. Contrary to the impairment when two cognitive tasks demanding top-down processing are performed simultaneously [37], we may speculate that self-paced exercise does not rely on top-down processing.

Alternatively, as a Reviewer of this manuscript pointed out, a potential dual-task effect could not be discarded [38] in our study, suggesting the involvement of top-down processing in both the cycling and n-back tasks. Indeed, there was a rather large difference in RT (30.46%) between the 2-back and the 1-back condition, which might be a sign of interference between the physical and cognitive task. For example, Jaeggi et al. [39] found that the difference in RT between the 2-back and 1-back tasks in dual-task conditions was 20.64%, whereas in single task conditions it was only 7.38%. The magnitude of the change in performance between conditions in our study was larger in the cognitive than in the physical task, which might suggest that, even though both tasks relied on top-down processes, participants prioritized the first over the latter. Nonetheless, this interpretation is speculative, as the purpose of our study was not to test potential dual-task effects, but to control for them [39].

There are, however, other possible explanation. We propose that despite the task was challenging enough, our sample of experienced cyclists (even though they were not elite cyclists) could have already automatized the self-paced effort so that they would not require high demands of top-down processing to self-regulate during the 20 min exercise [40]. Indeed, although there are not previous published attempts to measure the impact of cognitive load during self-paced cycling exercise, previous related findings would suggest that cyclists with a higher level of expertise might be more resistant to the negative effect of a high cognitive load [41]. In Martin et al.’s study, professional cyclists did not impair their performance in a 20 min time-trial after completing a 30 min mental exertion task (Stroop task) compared to a control condition. However, the group of recreational cyclists impaired their performance in the mental exertion condition and completed less correct answer in the Stroop task. Therefore, it would seem that there was also difference between trained and professional cyclists. However, the control condition in Martin et al.’s study did not involve any cognitive activity (10 min seated), so any effect of the cognitive task on exercise performance could have been due to the mere effect of doing a cognitive task versus doing nothing.

The present research needs to be considered in the context of some limitations. The Bayes factor indicated that there was an anecdotal evidence in favor of the alternative hypothesis. Therefore, if there was a small effect, we could not have been able to find with our sample size (n = 28). Additionally, the duration of the self-paced exercise and cognitive tasks could have not been long enough to induce a high cognitive load in trained cyclists. Finally, as noted above, the level of expertise might be a crucial factor to explain the effect of cognitive (executive) load on self-paced exercise.

## Conclusion

The effect of cognitive load on concurrent self-paced exercise performance is scarce, even if the role of cognition of exercise and sport performance is a current topic of debate. Our data appear to challenge the idea that self-paced exercise is regulated by top-down processing given that despite of the evident difference in cognitive load between the two conditions, participants did not seem to impair their performance. Our study, however, does not provide the definitive answer on whether self-pacing the physical effort rely on top-down processing, but opens new interesting avenues for future research on this topic that should consider factors like potential dual-task effects and sport expertise.
