# Correction: No evidence of the effect of cognitive load on self-paced cycling performance

**DOI:** 10.1371/journal.pone.0219340

**Published:** 2019-07-01

**Authors:** Darías Holgado, Mikel Zabala, Daniel Sanabria

In the Conclusions subsection of the Abstract, there is an error in the first sentence. The words “seem to” should not be bolded. The correct sentence is: Our data seem to challenge the idea that self-paced exercise is regulated by top-down processing, given that we did not show clear evidence of exercise impairment (at the physical, physiological and subjective levels) in the high cognitive load condition task with respect to the low working memory load condition.

## References

[1] Holgado D, Zabala M, Sanabria D (2019) No evidence of the effect of cognitive load on self-paced cycling performance. PLoS ONE 14(5): e0217825 10.1371/journal.pone.0217825 31150517 PMC6544270

